# Impedimetric Detection of Cancer Markers Based on Nanofiber Copolymers

**DOI:** 10.3390/bios14020077

**Published:** 2024-01-31

**Authors:** Noha Elnagar, Nada Elgiddawy, Waleed M. A. El Rouby, Ahmed A. Farghali, Hafsa Korri-Youssoufi

**Affiliations:** 1 Materials Science and Nanotechnology Department, Faculty of Postgraduate Studies for Advanced Sciences (PSAS), Beni-Suef University, Beni-Suef 62 511, Egypt; nohahamdy@psas.bsu.edu.eg (N.E.); waleedmohamedali@psas.bsu.edu.eg (W.M.A.E.R.); farghali@psas.bsu.edu.eg (A.A.F.); 2 Université Paris-Saclay, Centre National de la Recherche Scientifique (CNRS), Institut de Chimie Moléculaire et des Matériaux d’Orsay (ICMMO), ECBB, 17 Avenue des Sciences, Site Henri Moisson, 91400 Orsay, France; 3 Department of Biotechnology and Life Sciences, Faculty of Postgraduate Studies for Advanced Sciences (PSAS), Beni-Suef University, Beni-Suef 62 511, Egypt; n.giddawy@psas.bsu.edu.eg

**Keywords:** cancer, folate receptors, impedimetric nanofiber biosensor, electrochemical signal, bio-friendly polymers, medical industry

## Abstract

The sensitive determination of folate receptors (FRs) in the early stages of cancer is of great significance for controlling the progression of cancerous cells. Many folic acid (FA)-based electrochemical biosensors have been utilized to detect FRs with promising performances, but most were complicated, non-reproducible, non-biocompatible, and time and cost consuming. Here, we developed an environmentally friendly and sensitive biosensor for FR detection. We proposed an electrochemical impedimetric biosensor formed by nanofibers (NFs) of bio-copolymers prepared by electrospinning. The biosensor combines the advantages of bio-friendly polymers, such as sodium alginate (SA) and polyethylene oxide (PEO) as an antifouling polymer, with FA as a biorecognition element. The NF nanocomposites were characterized using various techniques, including SEM, FTIR, zeta potential (ZP), cyclic voltammetry (CV), and electrochemical impedance spectroscopy (EIS). We evaluated the performance of the NF biosensor using EIS and demonstrated FR detection in plasma with a limit of detection of 3 pM. Furthermore, the biosensor showed high selectivity, reliability, and good stability when stored for two months. This biosensor was constructed from ‘green credentials’ holding polymers that are highly needed in the new paradigm shift in the medical industry.

## 1. Introduction

According to the World Health Organization (WHO), cancer is considered the second leading cause of death in the world, accounting for 10.0 million deaths in 2020 [1,2]. Therefore, the sensitive detection of cancer is crucial due to the increase in the global burden of cancer. Various imaging techniques have been used for cancer diagnoses [3,4]. However, these techniques are time consuming; require sophisticated, expensive equipment and highly trained technicians for processing; and are a risk to human health due to radiation exposure [4]. Recently, they have been developed based on artificial intelligence and machine learning capabilities [5,6]. However, their development has been slowed by some systematic challenges, such as dataset availability, which is often the guidance for methods research rather than clinical relevance, and research incentives, such as optimization for publication [7]. Therefore, it is of the utmost importance to develop simple, safe, and cost-effective techniques for the early detection of cancer.

FRs are well known as a tumor biomarker and cell membrane glycoproteins. The overexpression of FRs in body fluids has been demonstrated in many studies, even though they are membrane-bound proteins [8,9]. This overexpression rate can vary depending on the tumor type and burden, but it is usually in the ng/mL range [10]. For example, in a recent study, the lower levels of FRs found in the plasma of ovarian cancer patients were 6.25 and 3.125 ng/mL, respectively [10]. In another recent study, the level of FRs in the serum of lung cancer patients was 0.4 ng /mL [11]. In addition, the migration of cancer cells into the circulatory system was observed in the metastasis stage of cancer, supporting the use of body fluids for cancer detection and their evolution during therapy [12]. FR plays a vital role in cell proliferation and endocytosis [13]. It is highly expressed on the surface of cancer cells and its expression is restricted in normal tissues. Therefore, it could be used as an important cancer biomarker and its level could be used to predict cancer stages [14,15]. FRs have been detected in tissue samples using different techniques, such as surface enhanced Raman spectroscopy (SERS) [16], proteomics assays [17], fluorescence imaging [18,19], and positron emission tomography (PET) [20,21]. However, these techniques are tedious, costly, time consuming, pose a risk to human health due to toxic radioligands, and have low sensitivity, such as FA-fluorescein isothiocyanate dye, which has a short systemic circulation time, and therefore does not target tumors efficiently [22].

Electrochemical biosensors can effectively overcome these drawbacks and address the need for the early detection of cancer biomarkers in biofluid samples due to their simplicity, low cost, high selectivity, and sensitivity with rapid, reliable responses [23,24]. In particular, electrochemical impedance spectroscopy (EIS) is a powerful label-free technique that can directly determine biomolecular recognition actions and the appropriate modification of sensitive elements on the electrode surface [25,26]. Compared to proteins, peptides, and antibodies, folic acid (FA) has a high affinity for binding FR with a K_d_~10**^−^**^9^ M [22,27]. This affinity is due to the presence of specific binding sites on the FA molecule that are complementary to the structure of FRs. When FRs come into contact with FA, they bind to these binding sites, forming a complex. This complex can then be detected using various methods, such as EIS. It is also nonimmunogenic, inexpensive, highly stable, and has a low molecular weight, making it easy to conjugate for a transducer. Therefore, it can act as a biorecognition element in FR detection using an electrochemical sensing platform. Various works have demonstrated that the interaction between FR and FA blocks the electron transfer through the insulating cell membrane, measured by following redox markers in the solution using cyclic voltammetry (CV) or electrochemical impedance spectroscopy (EIS) [28]. Bai et al. developed an electrochemical sensor based on reduced graphene oxide (rGO) nanosheets combined with FA to detect FR [28]. The response of the rGO–FA deposited onto a glassy carbon electrode (GC) sensor was evaluated using differential pulse voltammetry (DPV) by following the redox signal of [Fe(CN)_6_]^3−/4−^ [28]. Recently, Soares et al. electrochemically synthesized Poly(3,4-ethylenedioxythiophene) nanotubes (PEDOT-NTs) onto a stainless steel mesh electrode followed by the electrodeposition of gold nanoparticles (AuNPs) onto the surface of the nanotubes to detect the folate binding protein (FBP) using the avidin/biotin pair [29]. The electrochemical sensor based on a zirconium metal organic framework was utilized and modified with FA by Du et al. [13]. It was immobilized onto gold electrodes and used to detect a breast cancer cell line. Although the performance of these biosensors is promising, providing biosensors with high sensitivity, chemical reaction methodology, and biocompatible materials is still highly needed for biosensor implementation in diagnosis. Furthermore, the methods used in most of the literature for biosensor fabrication involved the covalent attachment of FA to the nanomaterials [30,31].

The study performed in this work developed an electrochemical biosensor based on a green procedure strategy to fabricate nanofibers based on biopolymers for the early detection of FRs in body fluids. The nanofiber was formed from a composite of biopolymer alginate with polyethylene oxide and FA as a bioreceptor using the electrospinning method.

Electrospinning is considered one of the most promising nanotechnology techniques due to its simplicity, versatility, low cost, and scalability [32]. Electrospun nanofibers have unique features compared to traditional screen-printed electrodes, as in [33,34], which enable the design of new biosensors with high sensitivity, selectivity, portability, and the ability to efficiently immobilize biomolecules, which is beneficial for biosensing applications. These characteristics include a high surface-to-volume ratio, porosity, permeability, malleability, mechanical properties, stability, versatility, scalability with a low cost, and ease of surface functionality modifications [35,36]. Alginate (Alg) is a marine-derived polysaccharide found in brown algae cell walls [37]. There is an unprecedented interest for using alginate in several biomedical applications that extends to the substitution of petroleum-derived polymers from an economical point of view [32,38]. This great interest is due to its up-and-coming features, such as biocompatibility, low cost, biodegradability, versatility, ease of functionalization, and excellent gel-forming capacity. Regarding the electrospinnability of alginate, its polyelectrolytic character, along with the lack of chain entanglement, gelation at low concentrations, and high surface tension, contribute to the limited spinnability of the (Alg) solution alone [39]. Therefore, we used PEO as a carrier polymer to facilitate the spinnability of Alg [40] and to prevent non-specific interactions [41]. 

**Scheme 1:**
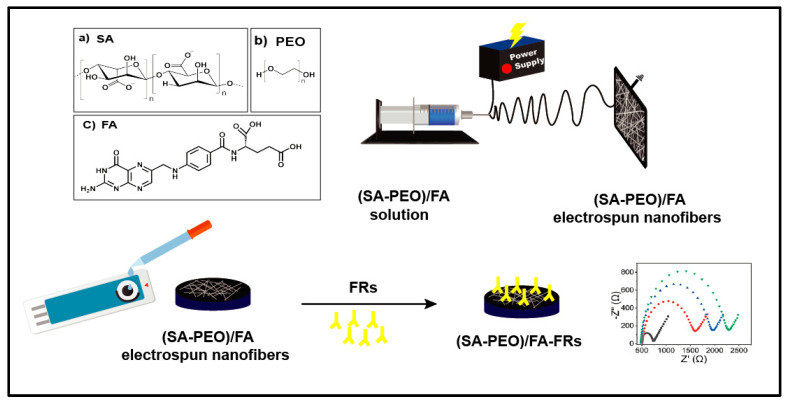
Schematic illustration of the fabrication process of the SA and PEO/FA nanofiber (NF) –based electrochemical biosensor for FR determination using the EIS technique. (**a**) the chemical structure of SA (sodium alginate), (**b**) The chemical structure of PEO (poly ethylene oxide), (**c**) The chemical structure of FA (folic acid).

Thus, we aimed to develop a biosensor based on a green procedure strategy to fabricate an NF structure based on biopolymers for the early detection of FRs in body fluids. The NF was formed from a composite of Alg, PEO, and FA as a bioreceptor in one step using the electrospinning method. The detection of the FRs was measured using EIS. This approach combined the advantages of SA and PEO as promising green supporting polymers and FA as a recognition biomolecule with a high binding affinity to the FRs. The electrospinning synthesis parameters were optimized, such as the applied voltage, concentration of various polymers, flow rate, and solution conductivity [42]. The obtained nanofiber morphology, chemical structure, and electrochemical properties were characterized using SEM, dynamic light scattering (DLS), FTIR, CV, and EIS. The biofilm was drop-cast on a screen-printed electrode (SPE) and the binding of the FRs to FA immobilized onto the surface was monitored using the EIS in the buffer and plasma (Scheme 1). We demonstrated that this strategy for biosensor construction allowed us to obtain a biosensor using a one-step synthesis, which could detect FRs with high sensitivity and specificity in a wide range of concentrations in plasma. This performance of NF-based biosensor [35,43] was impressive for the detection of biomarkers due to its large surface area, which allowed for a high loading of bioreceptors, and then an interaction with analytes, resulting in an ultrasensitive detection signal. The biosensor also presented outstanding reproducibility and stability, demonstrating the efficiency of the NF biosensor fabrication strategy, which opens the way for applications in various detection systems.

## 2. Materials and Methods

### 2.1. Materials 

The sodium alginate (SA) was purchased from BDH Laboratory Supplies Poole, BH15 1TD, England. Folic acid (FA) 98% was purchased from Loba Chemie PVT.LTD-Mumbai, India. The Human FOLR2 Protein (FRs) > 90% (Mw = 26.5 KDa) and Human Serum Albumin Protein (HSA) > 95% (Mw = 67.4 KDa) were purchased from ACRO Biosystems, Newark, DE, USA. The polyethylene oxide (PEO) (Mw = 600,000 Da), bovine serum albumin (BSA) < 96% (agarose gel electrophoresis), human plasma, Tris-HCl buffer, potassium chloride, and potassium ferricyanide/potassium ferrocyanide were purchased from Sigma-Aldrich Co. (St. Louis, MO, USA). All the other reagents were of analytical grade and were used without further purification. All the aqueous solutions were filtered using Milli-Q purified ultrapure water with a resistivity of 18.2 MΩ cm^−1^.

### 2.2. Instrumentation 

Electrospinning was carried out using the NANON apparatus from MECC Ltd. (Fukuoka, Japan) with an aluminum grounded collector plate. The solutions were electrospun through a 23-gauge needle. All the experiments were carried out at room temperature (22 to 26 °C) and with a relative humidity of 45 to 55%. 

The morphology and size of the NFs were evaluated using a Field Emission Scanning Electron Microscopy (FE-SEM) Model Quanta 250 FEG (field emission gun). The fiber diameter was estimated using the Image J (version: Java 1.8.0_345) (National Institutes of Health, Bethesda, MD, USA) analysis software. The zeta potential (ZP) measurements were performed at 25 °C using a Zetasizer Nano ZS 90 from Malvern Zies, sigma 500. The FTIR measurement was performed using a Bruker Vertex 70 instrument equipped with an ATR pike and an MCT detector. 

The EIS and CV measurements were carried out using a Metrohm Autolab PGSTAT12 Potentiostat controlled via the Nova software (1.10). Screen-printed carbon electrodes (SPEs) from Metrohm (dropsens) were used for the biosensor formation. The EIS measurements were performed in the frequency range of 0.1 Hz to 100 KHz with a modulation potential of 10 mV and the EIS data fittings were performed using the Nova software. 

### 2.3. Fabrication of NFs 

We optimized the preparation conditions of the SA-PEO/FA NFs through many experiments to obtain nanofibers with a high surface area and a high loaded FA bioreceptor. For this purpose, we optimized the synthesis of the NF polymer formed with SA-PEO by varying the ratio between each polymer. Thus, various ratios of the polymer mixture were studied. Firstly, 1.5 wt.% PEO powder (*w*/*v* in respect to the solvent) was dissolved in deionized water (solution A) under stirring at room temperature for 6 h. A total of 3 wt.% SA powder was dissolved in deionized water at room temperature under vigorous stirring for 20 min (solution B). Both solutions were dissolved separately until homogeneous solutions were obtained. Then, various mixtures of the two polymers (solution B and solution A; 3 wt.% SA, 1.5 wt.% PEO) were blended at a weight ratio of 70/30, 30/70, and 50/50, respectively, and investigated to form the SA-PEO NF. Electrospinning was then performed within the various solutions to obtain different nanofibers with various biopolymers. The electrospinning parameters were also optimized, and the polymer solutions were fed at 0.5 mL/h. An electric voltage of 15 kV was applied with a needle tip–collector distance of 13.5 cm (Table S1). For the NF formed and based on the effect of the ratio on the electrospinnability, the ratio of 30/70 (SA/PEO) was chosen (Table S1 raw F9) because these conditions gave high yields of nanofibers with a uniform morphology. This ratio and these conditions were maintained for NF formation using FA.

Regarding the FA association, initially FA was added to the mixture of SA. The PEO solution with a weight ratio of 30/70 was chosen based on the preliminary tests and was stirred overnight under dark conditions to obtain a homogeneous solution containing SA, PEO, and FA. NF formation using two different concentrations of FA (20 mg and 40 mg) was performed. The nanofibers SA/PEO/FA, formed within the two concentrations of FA, were formed through electrospinning. The SA/PEO/FA NFs with 20 mg of FA gave higher yields and a uniform morphology and were chosen for further study (Table S1, rows F10 and F11). 

We synthesized two NF composites, one composed of the two polymers SA and PEO (SA-PEO) and one formed by the two polymers SA and PEO and the bioreceptor FA (SA-PEO/FA). These two NFs were used to demonstrate the effect of the attachment of FA on the sensing ability of the FR proteins.

### 2.4. Preparation of the Biosensor and FR Biosensing Assay

The SA-PEO/FA NFs and SP-PEO-modified electrodes were prepared by drop casting 4 µL solution of a 1 mg mL^−1^ of NF suspension on the surface of the SPCE. The solutions were prepared by dispersion of 1mg of SP-PEO or SA-PEO/FA in 1 mL (D.I) with the aid of ultrasonication. The electrodes were dried at 50 °C for 30 min. For the bioassay study, the SA-PEO/FA NF-modified electrodes were incubated for 1 h at 4 °C with various concentrations of FRs from 0.1 pM to 100 nM diluted in 100 mM of Tris-HCl at pH 8.0, containing a 0.1% BSA to block the remaining active sites and eliminate the non-specific binding effect. The SPE/SA-PEO/FA electrode was then rinsed with Tris-HCl to remove any non-bonded FRs. The preparation process for the modified electrode is shown in Scheme 1.

The measurement of the biosensor’s response was performed in 5 mM [Fe(CN)_6_]^3−^/^4−^ mixed with a buffer solution before and after the FR attachment using EIS. The variation of the charge transfer resistance was obtained after fitting and the average variation was plotted with the concentration of the FR.

The calibration curve in the linear part allowed us to calculate the limit of detection (LOD) based on the IUPAC model, as shown in the following equation.

$$\mathrm{LOD}=\frac{3\times SD}{S}$$

where SD is the standard deviation of blank calculated for three different measurements performed in [Fe(CN)_6_]^3−^/^4−^ and S is the slope of the calibration curve.

#### Detection in Human Plasma

The detection of FRs in human plasma was performed by doping human plasma with various concentrations of FRs. Firstly, human plasma was diluted in deionized water to 5 mL, then the stock solution of the FRs was diluted using human plasma to prepare the highest concentration (750 nM) FR solution. Serial dilutions of this highest concentration were done using a human plasma solution to obtain various concentrations of FRs in the plasma samples. 

### 2.5. Analytical Procedure of the Electrochemical Biosensor 

The CV and EIS experiments were performed for the characterization of the SA-PEO/FA NFs electrodes layers using the [Fe(CN)_6_]^3−^/^4−^ solution. The CV experiments were performed at potentials ranging from −0.4 to 1.0 V and a scan rate of 0.05 V/s. EIS was performed at open circuit potential (OCP) using a 10-mV amplitude in the frequency range from 0.1 Hz to 100 kHz. The EIS measurement for the detection of FRs was also performed in [Fe(CN)_6_]^3−^/^4−^ mixed with 0.1 M KCl.

## 3. Results and Discussion

### 3.1. Preparation of SA-PEO/FA NFs

The synthesis process was performed through the mixture of various solutions, depending of the composition of NF, the mixture of the polymers SA and PEO and mixture of solution SA and PEO with FA, as shown in Scheme 1. PEO was used as a carrier polymer for alginate from the needle tip to the collector to facilitate its electrospinnability [36]. The NF copolymers formed with this SA-PEO/FA solution were obtained through electrospinning by mixing various solutions of polymers and the ratio was optimized to obtain nanostructured fibers with the desired diameter. Firstly, the electrospinning parameters, such as the potential, feed rate, and needle–collector distance, were studied in a mixture of the two polymers SA and PEO with a concentration ratio of SA and PEO of 30/70, as shown in detail in the Supporting Information. The NFs fashioned from the two polymers and FA bioreceptor was then formed (Table S1). Thereafter, we studied the structural and electrical properties of the two NFs formed. 

### 3.2. Morphological, Structural, and Electrochemical Characterizations of the Biosensor

#### 3.2.1. Morphological Characterization

**Figure 1:**
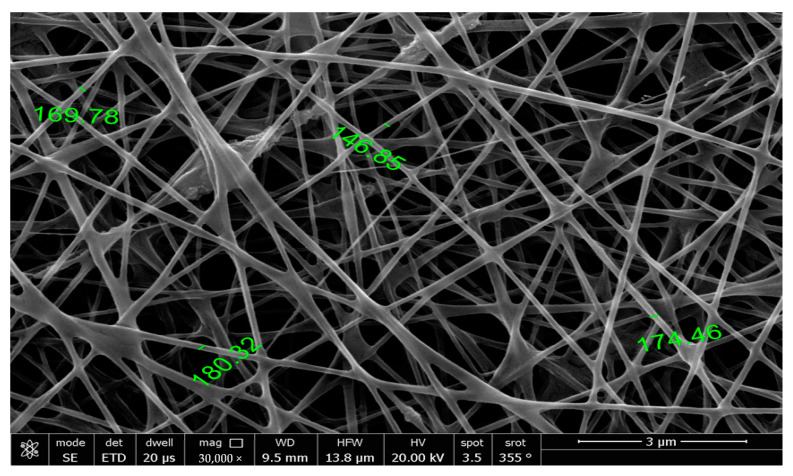
High-resolution scanning electron microscope (HRSEM) image of the nanofibers obtained from the aqueous solution of SA-PEO/FA.

The morphologies of the nanofibers were studied using SEM to determine their structures, diameters, and surface areas. Figure 1 shows the SEM image of the NF of SA-PEO/FA and the homogenous structure of the NF with smooth and uniform surfaces, revealing that the NFs had a well-regulated structure. The fibers had an average diameter of ca. 167 nm, which was smaller than previously reported due to the optimized conditions of electrospinning [44] and a surface area of 43.4 m^2^/g. This homogenous structure of NFs with small diameter confirmed the successful electrospinning process to produce NFs that enhanced the active surface area, making them advantageous as electrochemical biosensors for the early detection of cancer biomarkers.

#### 3.2.2. Structural Characterization 

**Figure 2:**
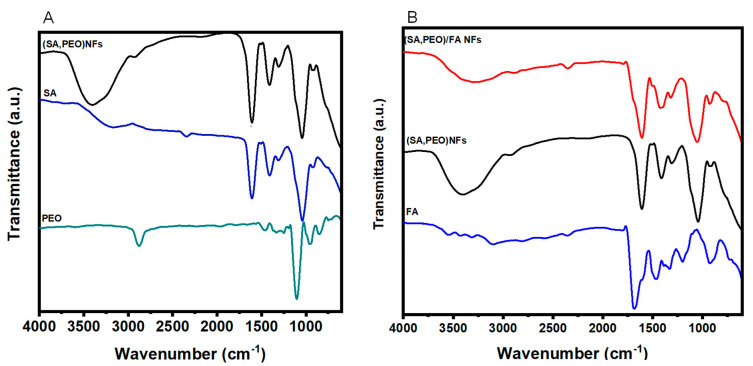
FTIR spectra (**A**) comparing the SA, PEO, and SA–PEO NFs and (**B**) comparing the FA SA–PEO/FA NFs and SA-PEO NFs.

The structural characterizations of the NFs were performed using FTIR to underline the chemical structure of the nanomaterials obtained after electrospinning and the efficient formation of NFs with FA association. The chemical structures of each component are presented in Figure 2A and the FTIR spectra of the NFs without and with a conjugation of FA are shown in Figure 2B.

Firstly the FTIR spectrum of the SA-PEO NFs were compared to individual polymers (Figure 2A) and showed bands corresponding to the two polymers with a small shift attributed to the potential hydrogen bonding between the etheric oxygen of PEO and the hydroxyl groups of SA [45]. As a result of the high SA-PEO content, most of the detected bands could be attributed to the backbone of these polymers. The spectrum of SA shown in Figure 2A displayed the broad band at 3700–3000 cm^−1^, attributed to the O–H stretching; the vibrations bands at 1598 and 1408 cm^−1^, which were assigned to the asymmetric and symmetric stretching vibrations of carboxylate (C=O) and C–O–C stretching at 1028 cm^−1^ [46,47]. However, on the other hand, the main characteristic bands of PEO could be seen in Figure 2A between 842 cm^−1^ and 1100 cm^−1^, corresponding to C–O–C bending and asymmetric stretching, and the other characteristic bands could be easily seen at 2930-2890 cm^−1^, arising from the vibration of CH. The peak that appeared at 1342 cm^−1^ was denoted as the O-H bending vibration in the literature [47,48]. 

The incorporation of FA into the SA-PEO/NF NFs showed a new band in the FTIR spectra (Figure 2B) centered at 1692 cm^−1^ and assigned to the carbonyl group of the of FA [28,49]. Another characteristic IR absorption band at 1484 cm^−1^ was due to the vibration of the phenyl ring [49,50,51]. The bands at 1193 and 1454 cm^−1^ in the FA spectrum were related to the CH bending vibrations of the p-aminobenzoic acid moiety [22,52]. Furthermore, it is noteworthy that there was a decrease in the SA broadband at 3700–3000 cm^−1^ after the incorporation of FA into the NFs. This could be attributed to the interaction between FA and SA molecules due to the potential hydrogen bonding. 

The zeta potential measurements were performed to analyze the modification of the NF surface for the two NFs formed. Table 1 shows the modification of the surface charge for the NF formed without FA and with an association to FA. The charge SA-PEO/FA NFs conjugated with FA made the zeta potential of the NFs more negative, which confirmed the successful conjugation of FA into SA-PEO NFs. The zeta potential curves of the various NFs can be seen in Figure S1.

#### 3.2.3. Electrochemical Characterization of SA-PEO/FA NFs

**Figure 3:**
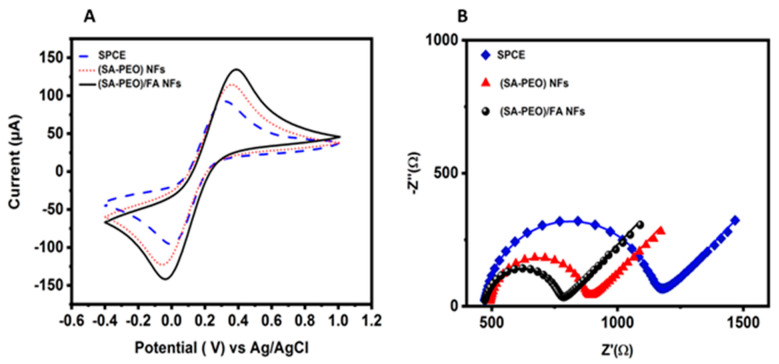
(**A**) CV curves in the [Fe(CN)_6_]^3−^/^4−^ redox probe of the SPCE and modified SPE SA-PEO vs. SA-PEO/FA at potentials ranging from −0.4 to 1.0 V and a scan rate of 0.05 V/s; (**B**) EIS in the [Fe(CN)_6_]^3−^/^4−^ redox probe of the SPCE and modified SPE SA-PEO vs. SA-PEO/FA obtained at the OCP with a DC of 10 mV and a frequency range of 100 kHz to 0.1 Hz.

Electrochemical characterization was performed using CV and EIS to analyze the electrical behavior of each layer. The analyses were performed in the [Fe(CN)_6_]^3−^/^4−^ redox marker. The CV data obtained with the modified SPE with two NFs, SA-PEO and SA-PEO/FA, showed an increase in the redox current compared to SPCE, demonstrating the high surface area of the NFs (Figure 3A). When FA was introduced to the NF, the electroactivity also increased. This result demonstrated an electroactivity increase in the layer formed by the SA-PEO/FA NF due to the chemical properties of FA. 

We also recorded the CV signals at varying scan rates from 10 to 100 mVs^−1^ using SPCE and modified them with SA-PEO NFs and SA-PEO/FA NFs (Figure S2A–C). We analyzed the effect of the scan rate on the intensity of peak current. A linear relationship between the square root of the scan rate and the peak current (Figure S2D–F), which ensures the diffusion-controlled electrochemical process, were observed. This property is highly needed for electrochemical affinity biosensors. We calculated the active surface area of each layer of biosensor from the slope of the plot of the peak current versus the scan rate.

To estimate the electroactive surface area of all the modified electrodes, we employed the Randles–Sevcik equation (Equation (1)).

Ip = 2.69 × 10^5^ n^3/2^ A D^1/2^ C v^1/2^ (1)

where n is the number of electrons that takes place in the redox reaction, D is the diffusion coefficient, v^1/2^ is the square root of the scan rate, and Ip is the peak current. The surface area (A) can be determined from the slope between Ip and v^1/2^ (Figure S3). A higher slope refers to a larger active surface area, which confirms that more electroactive sites are available for the interaction between FA and FRs, and therefore enhances the sensitivity of the biosensor. Using Equation (1), we calculated the electroactive surface area of the bare electrode as 10.2 mm^2^, the SA-PEO NFs as 13.5 mm^2^, and the SA-PEO/FA NFs as 14.8 mm^2^ (Table 2). Our findings demonstrated the high conducting surface area of SA-PEO/FA NFs compared to the other modified surfaces. This underlined that the combination of biopolymers and FA leads to the formation of NFs SA-PEO/FA with a higher surface area and increased porosity.

EIS was performed in the same electrolyte to underline the electrical properties of the biolayers. The Nyquist plots showed a decrease in the semicircle from the SPCE compared to the SPE modified with NFs (Figure 3B). This variation demonstrated a decrease in the charge transfer resistance. To confirm this behavior, the EIS data were fitted with Randels equivalent circuit model (Table S2). The values showed a decrease in the charge transfer resistance from 674 Ω for the non-modified SPE to 381Ω and 298 Ω for, respectively, the SPE modified with SA-PEO and SA-PEO/FA NFs (Table 2). This behavior demonstrated the ionic conductivity of the biolayers when the three components SA, PEO, and FA formed NFs.

The Rct was related to the speed of the electron transfer between the redox molecules in the solution and the biolayer surface. For the redox process in the solution, it was directly connected to the kinetic of the heterogenous electron transfer rate constants (k_0_) for the redox species. Thus, k_0_ could be calculated from the Rct data. This parameter allowed us to compare the electron transfer ability for the various nanofibers with the bare electrode. 

When the EIS was measured for open circuit potential, a low potential was flowing and k_0_ could be determined from the Butler–Volmer equation with the current exchange i_o_ via Equation (2) [53] (more detail about the calculations are provided in the Supporting Materials).

(2) $$R_{CT}=\frac{RT}{n^{2}F^{2}ACk_{0}}$$

where *n* is the number of electrons in the redox reaction; *F* is Faraday’s constant, 96,485 C/mol; *A* is the surface area of the working electrode in cm^2^; C is the concentration of the redox species in mol/cm^3^; *R* is the gas constant 8.3144 J/molK; and *T* is the temperature in K.

The results showed an increase in k_0_ from 1.48 × 10^−3^ k^0^/cm s^−1^ for the bare electrode to 2.61 × 10^−3^ k^0^/cm s^−1^ and 3.35 × 10^−3^ k^0^/cm s^−1^ for, respectively, the SA-PEO and SA-PEO/FA NF-modified SPEs (Table 2), demonstrating a faster electron transfer ability in the modified NF SPE. Collectively, these results demonstrated that the electron transfer ability was higher with the NFs formed from biopolymers compared to the bare SPE. They offer a comprehensive view of the electrochemical characteristics and the efficiency of the SA-PEO/FA NFs as a potential FR biosensor due to the porosity and high permeability of the biolayer, which meet the specific needs of the electrochemical sensing properties. 

##### Biosensors Optimization

Optimization under the conditions of the biosensor formation played a key role in the analysis performance. In our work, the concentration of FA was optimized to achieve the best sensing performance. The concentration of FA during electrospinning was then varied from 40 to 20 mg.

When the concentration of FA in the polymeric solution was higher than 20 mg, the electrospinning process was hindered and no NFs were obtained. NF formation was largely governed by the surface charge of the polymeric solution, and therefore its conductivity. The negative charge of FA due to the carboxyl groups in its structure [22,54,55] increased the conductivity of the polymer solution beyond the critical value due to the significant increase in the negative charge density on the surface of the polymer droplet. This led to a high repulsion force between the negative SA-PEO polymer droplets and FA, which hindered the electrospinning process [42,56].

Therefore, we prepared 1 g of a NF sheet containing 20 mg of FA. Furthermore, we optimized the amount of the nanocomposite and the FA in the biolayer by testing the electrochemical CV analysis technique. We investigated various concentrations of NFs to obtain various amount of FA. Two solutions were studied for the biosensor formation of the NF solution (1 mg·mL^−1^, where the FA amount was 0.02 mg, and 0.5 mg·mL^−1^ NFs with 0.01 mg of FA in the composite). A higher concentration was not tested because we obtain high viscosity of the solution. After drop casting the NFs onto the SPE, the CV in the redox marker was recorded and showed that the NFs formed with high concentrations led to improved electroactivity (Figure S2). This was related to the improved conductivity and surface area of the NFs formed with higher concentrations. A total of 1 mg·mL^−1^ of NFs was selected for the sensing measurements due to the higher concentration of FA and the improved active surface area.

### 3.3. Biosensors Properties Regarding FRs

**Figure 4:**
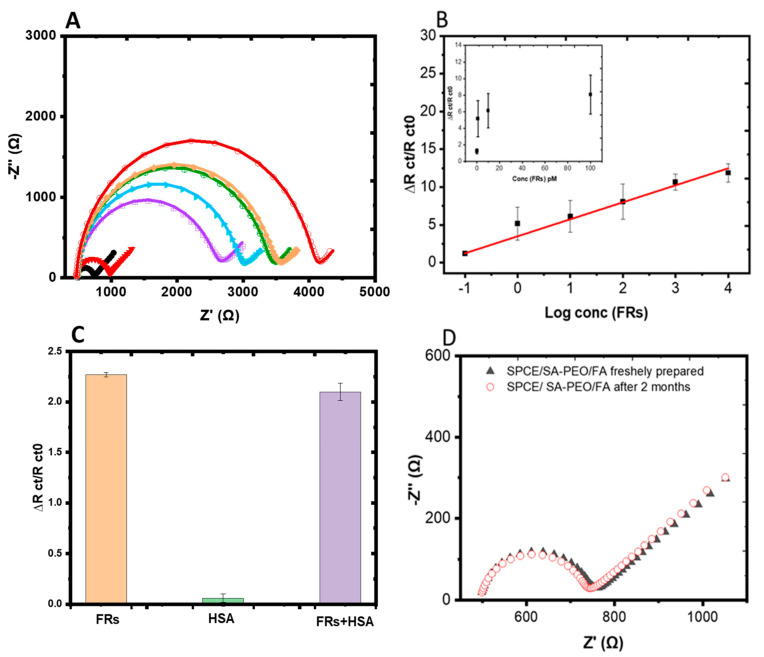
(**A**) Nyquist diagrams of a SA-PEO/FA-modified SPCE obtained with increasing concentrations of FRs by decade from 0.1 pM to 100 nM. The different colors refer to different concentrations of FRs, black is biosensor without FR, red: 0.1 pM, violet: 1 pM, blue: 10 pM, green: 100 pM, yellow: 1 nM, dark red: 10 nM (**B**) Calibration curve corresponding to the average variation of ΔRct/Rct_0_ versus the log concentrations of the FRs measured and inset with the linear part. (**C**) Histogram of the interference analysis of the NF biosensor after incubation with10 nM FRs, 100 nM HSA, and a mixture of both proteins 10 nM FRs+ 100 nM HSA. (**D**) Stability analysis of the NF biosensor using [Fe(CN)_6_]^3−^/^4−^ after storing for two months at 4 °C. EIS was measured at the OCP with a DC of 10 mV and a frequency range of 100 kHz to 0.1 Hz.

The electrochemical response of the SA-PEO/FA NFs towards FRs was evaluated using FR solutions in the concentration range of 0.1 pM–10 nM. After the formation of the complex SA-PEO/FA/FR, the electrode was washed to remove non-attached FR. The response of the biosensors was monitored using EIS in the solution containing the redox marker [Fe(CN)_6_]^3−^/^4−^. The Nyquist plot showed the modification of the semicircle after the complex formation on the biolayer surface after each concentration (Figure 4A). This highlighted that the electrical properties of the sensing NFs, such as the charge transfer, resistance, and capacitance, were affected by the complex formation and the presence of FRs on the biolayer surface preventing the charge transfer to electrode. Fitting using the Randles circuit was performed through a modification of the capacitance by a constant phase element due to the inhomogeneous distribution of the charge related to the NFs structure (Table S3). The fitting data showed an increase in Rct with the FRs concentration. The variation Rct values obtained from the equivalent circuit model after FR detection were used for the generation of the calibration curve. The average variations of Rct were plotted with the logarithm of the FRs concentrations (Figure 4B) and showed a dynamic range for large concentrations. The limit of detection (LOD) was calculated at 2.5 pM from the slope of the linear part using the standard deviation of the blank test obtained for the measurement of independent modified electrodes (Table S4). 

These results were comparable with the early reported DNA, gold, and carbon material-based biosensors for FR detection, measured using the CV or DPV techniques (Table 3). In our study, we used a simple green synthesis method formed by biopolymers to construct the NF biosensor. The use of the biopolymer in this biosensor for point-of-care cancer diagnosis was compatible with the environmental concerns for the use of eco-friendly and sustainable technologies that make an effort towards a more responsible approach to healthcare. The NFs structure obtained by electrospinning offer a one-step synthesis of the biosensor, with high surface area and porosity with the ability of attaching FR biomarkers with a low detection limit and a high dynamic range. On the other hand, the impedimetric biosensor that was developed could directly determine biorecognition events at the electrode surface with a high sensitivity and a rapid and reliable response [26,57], making it ideal for point-of-care devices. 

### 3.4. Selectivity of the Biosensor

The selectivity of the SA-PEO/FA NF-based biosensor was proved using the EIS technique by incubating the modified electrodes with HSA, FR, or a mixture of the (FRs + HSA) solutions at a fixed concentration of 10 pM FRs vs. 100 pM HSA, as shown in the histogram (Figure 4C). The impedimetric response of the biosensor was much greater for the FR solution and was not affected by the presence of HSA in the mixture of the two proteins (Table S4). This was due to the specific interaction between FA and the FRs, resulting in a significant change in the impedance of the electrode, which was detected by the biosensor. In contrast, HSA, which is a non-target protein, did not bind to the FA groups on the biosensor surface. Therefore, the presence of HSA in the sample did not interfere with the detection of FRs. These findings demonstrated a high selectivity and reliability of the biosensor fabricated for FR detection even in complex biological samples in the presence of interfering serum proteins, such as HSA, and therefore demonstrated its selectivity for FR protein cancer marker.

### 3.5. Reproducibility, Reusability, and Stability of the Biosensor

The reproducibility was tested using four prepared fresh electrodes and the Nyquist plots showed the same behavior (Figure S5). The reproducibility of the proposed biosensor was examined in both Tris-HCl and human plasma using four independent electrodes in plasma under the same conditions as Tris-HCl. For this purpose, four parallel experiments for FR detection were performed in the concentration range of 0.1 pM–100 nM for the FRs. The biosensor exhibited an excellent reproducibility, as shown in Tables S3–S6, which showed the mean value of ∆(RCT) and the standard deviation (SD) calculated between the electrodes in Tris-HCl and plasma, respectively. It is worth mentioning that we took specific measures to achieve the reproducibility in our study, starting with producing NFs with a reproducible surface area and a ratio of components, which was an important challenging aspect due to the complex nature of the process and its sensitivity to various parameters such as the voltage, solution flow rate, and spinning time, as well as the material properties, environmental conditions, and characterization techniques that all enhanced the reproducibility. By fixing each parameter and working with a fresh solution, the reproducibility of the nanofibers was obtained from batch to batch.

Regarding reusability, the SA-PEO/FA NFs biosensor was reusable during the study since it was subjected to multiple cycles of FR detection and regeneration. After each cycle, the biosensor could be regenerated by washing it with a suitable Tris-HCl buffer solution to remove any non-specifically bound molecules. The regenerated biosensor could then be used for the next cycle of FR detection. The biosensor’s reusability was a significant advantage, as it allows for multiple measurements to be performed using the same biosensor, reducing the cost and time associated with fabricating new biosensors for each measurement.

Concerning the stability, the NFs powder or solution was stored fresh at 4 °C for 9 months without any modifications to the chemical properties or sensing ability. When the sensors were deposited onto the surface of the SPE, stability tests were performed by storing the modified electrode at 4 °C for two months. The EIS response was recorded after deposition and after two months (Figure 4D, Table S5). The result showed that the same signal was obtained without any variation from the freshly prepared one, confirming the good stability of the biolayer.

### 3.6. Detection in the Plasma Samples

**Figure 5:**
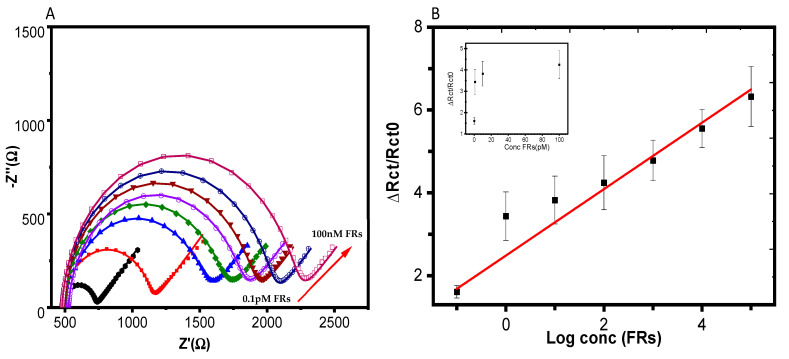
(**A**) Nyquist diagrams of the SA-PEO/FA-modified SPE obtained after association with FRs in human plasma with various concentrations from 0.1 pM to 100 nM, varying the concentration by decade. The black line refers to the biosensors free of FR and the different color lines refer to different FRs concentration incubated as following (0.1 pM, 1 pM, 10 pM, 100 pM, 1 nM, 10 nM, 100 nM). (**B**) Calibration curves corresponding to the average variation of ΔRct/Rct_0_ versus the log concentrations of the FRs measured using four independent electrodes. The inset presents the variation with the concentration. Note: the arrow represents the direction of variation of the Nyquist plot regrading increasing concentrations of FRs.

We explored the application of our biosensor in biological fluids, such as human plasma. In this case, the FRs in the concentration range (0.1 pM–100 nM) diluted in human plasma containing 0.1% BSA were analyzed. The Nyquist plots for the SPE incubated in human plasma showed the same variation compared to EIS, where the associations were performed in a buffer. When the concentration of FRs increased during incubation, the biosensor showed a large increase in the diameter of the semicircle with the FR concentration due to the binding of FRs to the SA-PEO/FA NFs (Figure 5A). This binding event created a barrier to the transfer of electrons between the electrode and the electrolyte, resulting in an increase in the charge transfer resistance. The curves exhibited a semicircle, as observed in the case of the Tris-HCl solution and the curves were fitted with the same equivalent circuit model to extract the values of the charge transfer resistance (Table S6). These values were used to quantify the binding of FRs to the SA-PEO/FA nanofibers. The calibration curve showed a linear relationship with the logarithm of the FR concentration (Figure 5B). The LOD obtained with the same method was 3 pM, which was nearly close to that in the Tris-HCl solution, as the LOD was 2.5 pM due to low effect of the plasma matrix. This low effect was provided by the NF composition where PEO prevented non-specific interactions that could be obtained with the plasma components. The value of the LOD obtained in plasma was very low compared to the reported results in the literature, confirming the competitive sensing performance of the SA-PEO/FA NFs and their reliability for FR detection. 

### 3.7. Risk Scenario Related to the Process, Sampling, and Detection

It was essential to consider the risks which could affect the mat’s performance. These risks were related to the process, such as the contamination of the NFs during fabricating and handling and the inconsistent manufacturing of the surface area, porosity, and fiber diameter due to errors in the fabrication process. The risks related to sampling included the improper handling of the sample, and the risks related to detection included measurement errors, the improper calibration of the instruments, and variations in the redox sample concentrations during the measurement as well as the skill of operators. These risks could affect the performance outcomes, such as the sensitivity, which may not be sufficient to detect low levels of analytes; the reproducibility, which yields consistent results when repeated; and the shelf-life, since NFs may degrade due to aging or exposure to chemicals. The strategies to mitigate such risks are process control, representative sampling, proper sample handling, instrument calibration, operator training, and method validation [63]. The processes performed during our experiments should be generalized to the sensor developments to avoid risk. 

Regarding the biosensor composition and fabrication method, the minimization of risk came from the synthesis approach. Only two steps for sensor fabrication were needed: one step for synthesis and one step for deposition on the SPE. Additionally, the approach for biosensor formation after optimization, including in NFs, all the components needed for biosensor design: the biopolymer PEO that prevented non-specific interactions, and the bioreceptor for the recognition element, had low risk. The introduction of BSA after SPE biolayer modification for blocking the remaining pinhole sites allow to avoid non-specific interactions and improve the detection in reel samples, such as plasma.

Electrospun nanofibers have unique features compared to traditional methods with various functionalization steps on the surface of screen-printed electrode. The biosensor design with one-step synthesis lead to high sensitivity, selectivity, portability, stability, and low-risk scenarios.

## 4. Conclusions

In summary, we proposed a simple way for selective, sensitive, and stable impedimetric biosensors to determine FRs using nanofibers formed by electrospinning. The synthesis methodology was optimized to improve the structure of nanofibers and the performance of the biosensors, starting with an aqueous polymeric solution that was based, for the first time, on a blend of SA, PEO, and FA to obtain SA-PEO/FA NFs. The biosensor combined the merits of the simple immobilization of bioreceptors and a NF structure, which enhanced the detection ability by amplifying the electrochemical signal and exhibiting a wide linear range and a low LOD. This was due to the low resistance to electron transfer and the large surface area of NFs, which enhanced the detection ability by amplifying the electrochemical signal. In addition, the selectivity was proven in the presence of the other interfering proteins and through detection in the reel sample, such as the plasma sample. Furthermore, the proposed biosensor has many advantages, such as being eco-friendly and its recyclability as it was formed from biopolymers. The biosensors involved an easy way for bioreceptor immobilization that afforded high stability. We anticipate that our strategy can help develop many NF-based biosensors by tailoring bioreceptors, which holds great promise for becoming the major tool for the ultrasensitive early detection of various biomarkers in biological fluids.

## Figures and Tables

**Table 1:** Zeta potential measurements of (SA-PEO) and (SA-PEO)/FA NFs.

Formulations	Zeta Potential (mV)	St Dev (mV)
NFs SA-PEO	−21.4	±3.80
NFs SA-PEO/FA	−33.2	±7.86

**Table 2:** Electrical and electrochemical properties of the NFs modified SPCE electrodes.

Element	Rct (Ω)	Ks (k^0^/cm s^−1^)	Electroactive Surface Area
SPCE	674.76	1.48 × 10^−3^	10.2 mm^2^
SPCE/SA-PEO NFs	381.96	2.61 × 10^−3^	13.5 mm^2^
SPCE/SA-PEO/FA NFs	298.07	3.35 × 10^−3^	14.8 mm^2^

**Table 3:** Comparison of electrochemical sensing of FRs using DNA, gold, carbon materials, and the NFs from our study.

**Sensing Material**	**Working Electrode**	**FR Range of Detection**	**Limit of Detection** **(FR Conc)**	Method	**Reference**
PDA–PEG–FA	ITO	FR (1 × 10^−3^–5 × 10^2^ ng/mL)	(2 × 10^−4^ ng/mL)	EIS	[58]
FA-DNA	Au	FR (1.0–20.0 ng/mL)	0.3 ng/mL	CV	[59]
PNT–FA	G	FR (8–13 nM)	8 nM	CV	[60]
DNA–FA	Au	FR (0.66–26.31 nM/L)	0.1204 nM/L	CV	[61]
PAH/FA	ITO	FR (10–40 nM)	0.7 nM, 1.5 nM	CV, EIS	[22]
rGO/FA-Py	G	FR (1–50 nM)	3.07 nM	CV	[62]
SA-PEO/FA NFs	SPE	FR (0.1 pM–100 nM)	2.5 pM	EIS	This work

## Data Availability

Data are contained within the article.

## Supplementary Materials

The following supporting information can be downloaded at: https://www.mdpi.com/article/10.3390/bios14020077/s1, Figure S1. The zeta potential curves for each layer of the nanofiber biosensor: (a) SA-PEO and (b) SA-PEO/FA. Figure S2. CV measured in [Fe(CN)_6_]^3-/4^- redox probe measured with various scan for SPE (A) and modified electrode with NFs SA-PEO (B) and SA-PEO/NF (NF). Scan from 10mv to 100mV/s; Corbe C, D and E representing the recorded of peak current variation versus wit root scan rate. Figure S3. The electroactive surface area of each layer of the nanofiber biosensor: (a) bare electrode, (b) SA-PEO-modified SPE, and (c) SA-PEO/FA evaluated by CV. Figure S4. Nyquist diagrams of a SA-PEO/FA-modified SPE obtained from increasing concentrations of FRs in 100 mM Tris-HCl at pH 8.0 for the remaining four electrodes. Figure S5. Nyquist diagrams of a SA-PEO/FA-modified SPE obtained from increasing concentrations of FRs in plasma for the remaining three electrodes. Table S1. Composition of the various solutions that were used for the preparation of nanofibers and the optimization of the electrospinning process. Table S2. The electroactive surface area and electron transfer rate constants (Ks) of the bare electrode, SA-PEO NFs, and SA-PEO/FA NFs. Table S3. Values obtained from the equivalent circuit elements by fitting the EIS experimental data of the five electrodes in Tris-HCl. Table S4. The selectivity of the developed biosensor for FRs by fitting the EIS experimental data in [Fe(CN)_6_]^3−^/^4−^ after incubating the NF-modified electrodes with 10 nM FRs (target) for 1 h, 100 nM HSA for 1 h, and a mixture of both proteins (10 nM FRs + 100 nM HSA) for 1 h. Table S5. The stability of the NF biosensor by fitting the EIS experimental data in [Fe(CN)_6_]^3−^/^4−^ after storing the NF-modified electrode for two months at 4 °C. Table S6. Values obtained from the equivalent circuit elements by fitting the EIS experimental data of four electrodes in human plasma. Determination of constant of heterogenous electron transfer from Rct.
