# Supplementary material for: Impedimetric Detection of Cancer Markers Based on Nanofiber Copolymers
Source: Biosensors (Basel). 2024 Jan 31;14(2):77. doi: 10.3390/bios14020077 (PMC10887276; doi:10.3390/bios14020077)
Supplement: Supplementary file 1 [file biosensors-14-00077-s001.zip › biosensors-2798156-supplementary.pdf]

Supplementary Materials

Impedimetric Detection of Cancer Markers Based on Nanofiber Copolymers

Noha Elnagar [1,2], Nada Elgiddawy [3], Waleed M. A. El Rouby [1], Ahmed A. Farghali [1] and Hafsa Korri-Youssoufi [2,*]

[1] Materials Science and Nanotechnology Department, Faculty of Postgraduate Studies for Advanced Sciences (PSAS), Beni-Suef University, Beni-Suef 62 511, Egypt
[2] Centre National de la Recherche Scientifique (CNRS), Institut de Chimie Moléculaire et des Matériaux d'Orsay (ICMMO), Université Paris-Saclay, ECBB, 17 Avenue des Sciences, Site Henri Moisson, 91400 Orsay, France
[3] Department of Biotechnology and Life Sciences, Faculty of Postgraduate Studies for Advanced Sciences (PSAS), Beni-Suef University, Beni-Suef 62 511, Egypt
* Correspondence: hafsa.korri-youssoufi@universite-paris-saclay.fr; Tel.: +33 6 23 94 90 28

Table of Contents:

List of Figures:

Figure S1. The zeta potential curves for each layer of the nanofiber biosensor: (a) SA-PEO and (b) SA-PEO/FA.

Figure S2. CV curves in the $[Fe(CN)_6]^{3-/4-}$ redox probe of the modified SPE SA-PEO/FA 0.01 mg vs. SA-PEO/FA 0.02 mg at potentials ranging from -0.4 to 1.0 V and a scan rate of 0.05 V/s.

Figure S3. The electroactive surface area of each layer of the nanofiber biosensor: **(a)** bare electrode, **(b)** SA-PEO-modified SPE, and **(c)** SA-PEO/FA evaluated by CV.

Figure S4. Nyquist diagrams of a SA-PEO/FA-modified SPE obtained from increasing concentrations of FRs in 100 mM Tris-HCl at pH 8.0 for the remaining four electrodes.

Figure S5. Nyquist diagrams of a SA-PEO/FA-modified SPE obtained from increasing concentrations of FRs in plasma for the remaining three electrodes.

List of Tables:

Table S1. Composition of the various solutions that were used for the preparation of nanofibers and the optimization of the electrospinning process.

Table S2. The electroactive surface area and electron transfer rate constants (Ks) of the bare electrode, SA-PEO NFs, and SA-PEO/FA NFs.

Table S3. Values obtained from the equivalent circuit elements by fitting the EIS experimental data of the first electrode in Tris-HCl.

Tables S.3b, S.3c, S.3d, and S.3e, respectively. Values obtained from the equivalent circuit elements by fitting the EIS experimental data of the four remaining electrodes in Tris-HCl.

Table S4. The selectivity of the developed biosensor for FRs by fitting the EIS experimental data in $[Fe(CN)_6]^{3-/4-}$ after incubating the NF modified electrodes with 10 nM FRs (target) for1 hr, 100 nM HSA for 1hr, and a mixture of both proteins (10 nM FRs+ 100 nM HSA) for 1hr.

Table S5. The stability of the NF biosensor by fitting the EIS experimental data in $[Fe(CN)_6]^{3-/4-}$ after storing the NF modified electrode for two months at 4 °C.

Table S6. Values obtained from the equivalent circuit elements by fitting the EIS experimental data of the first electrode in human plasma.

Tables S.6b, S.6c, and S.6d, respectively. Values obtained from the equivalent circuit elements by fitting the EIS experimental data of the three remaining electrodes in human plasma.

Determination of the constant of heterogenous electron transfer from Rct.

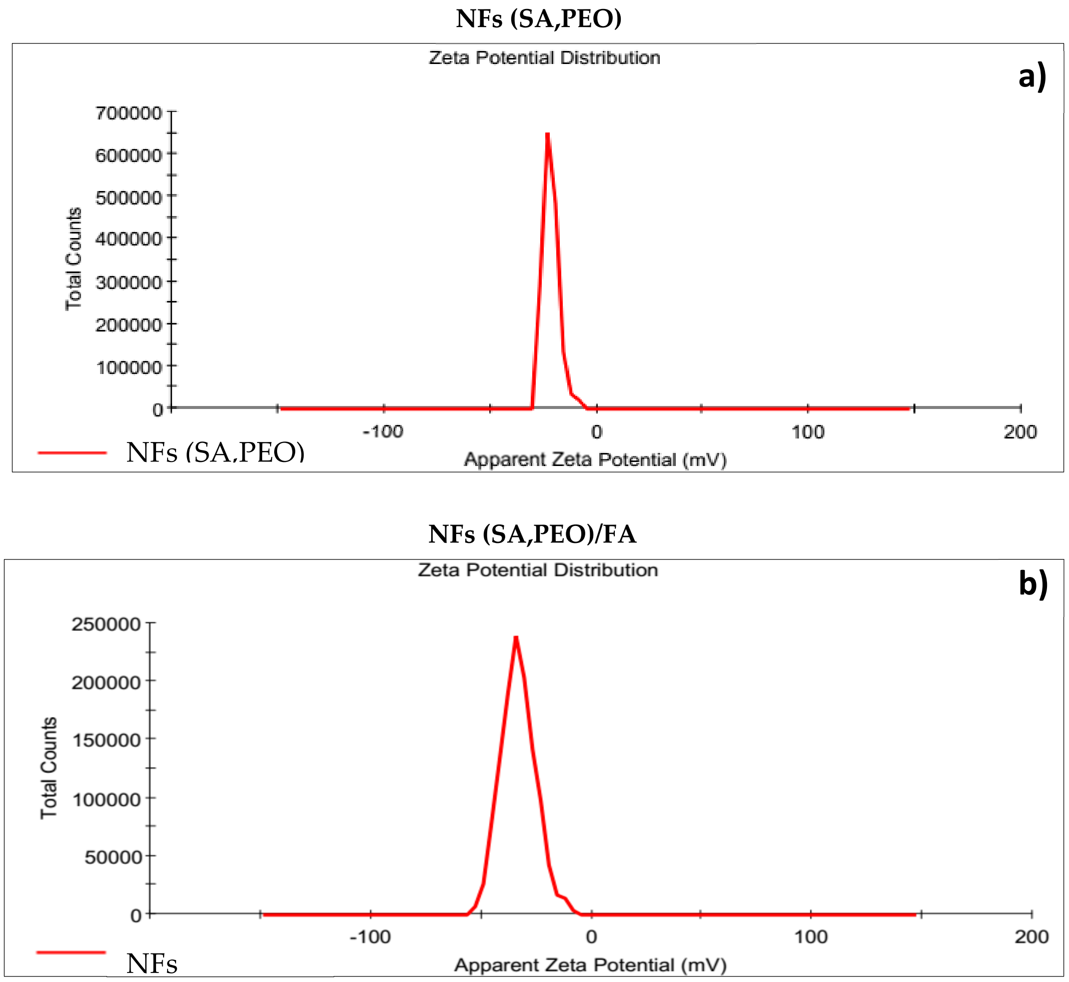

Figure S1. The zeta potential curves for each layer of the nanofiber biosensor: (a) SA-PEO and (b) SA-PEO/FA.

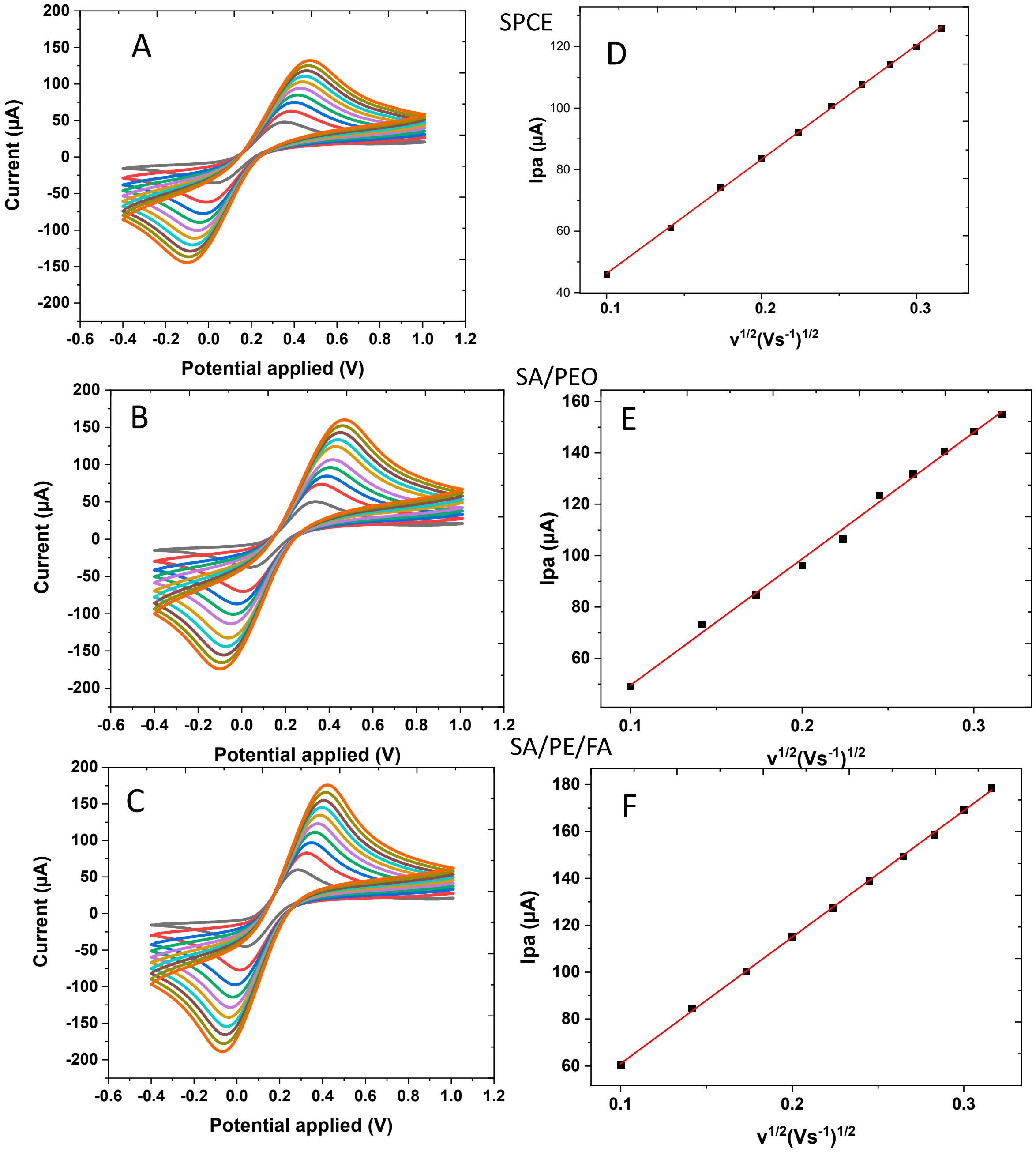

Figure S2. CV measured in the $[Fe(CN)_6]^{3-}/^{4-}$ redox probe measured with various scans for the SPE (**A**) and a modified electrode with SA-PEO (B) and SA-PEO/NF NFs. Scan from 10mv to 100mV/s; Graphs C, D, and E represent the recorded of the peak current variation versus the wit root scan rate.

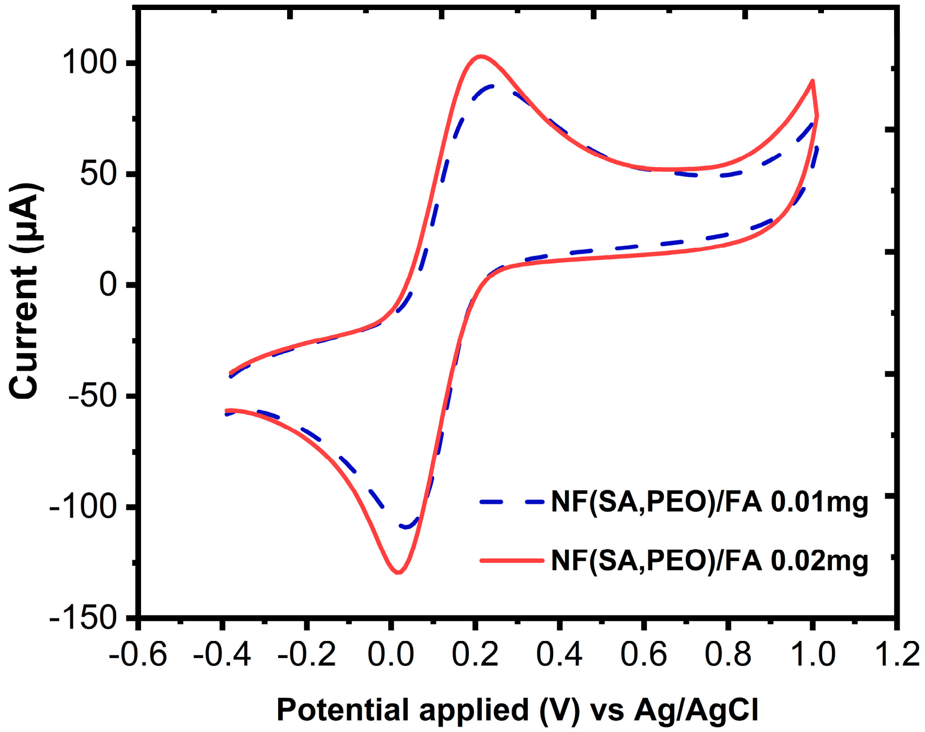

Figure S3. CV curves in the $[Fe(CN)_6]^{3-/4-}$ redox probe of the SA-PEO/FA-modified SPE 0.01 mg vs. SA-PEO/FA 0.02 mg at potentials ranging from -0.4 to 1.0 V and a scan rate of 0.05 V/s.

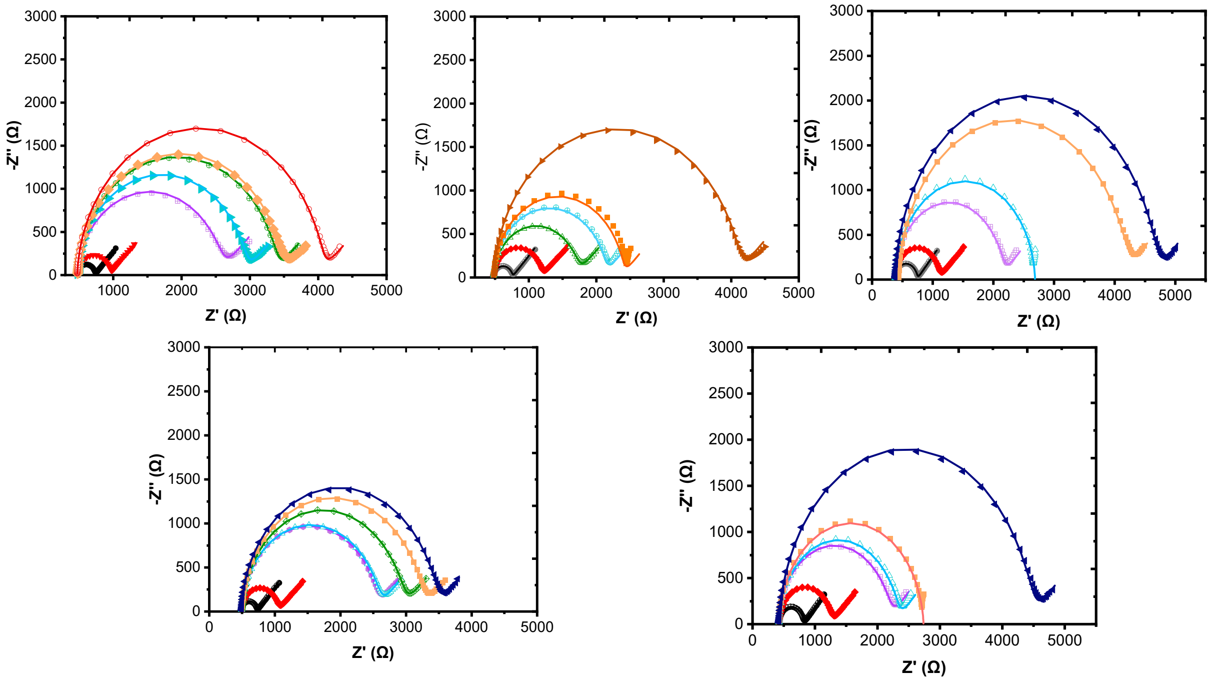

Figure S4. Nyquist diagrams of five biosensors from the SA-PEO/FA-modified SPE obtained from increasing concentrations of FRs in 100 mM Tris-HCl at pH 8.0 for five electrodes.

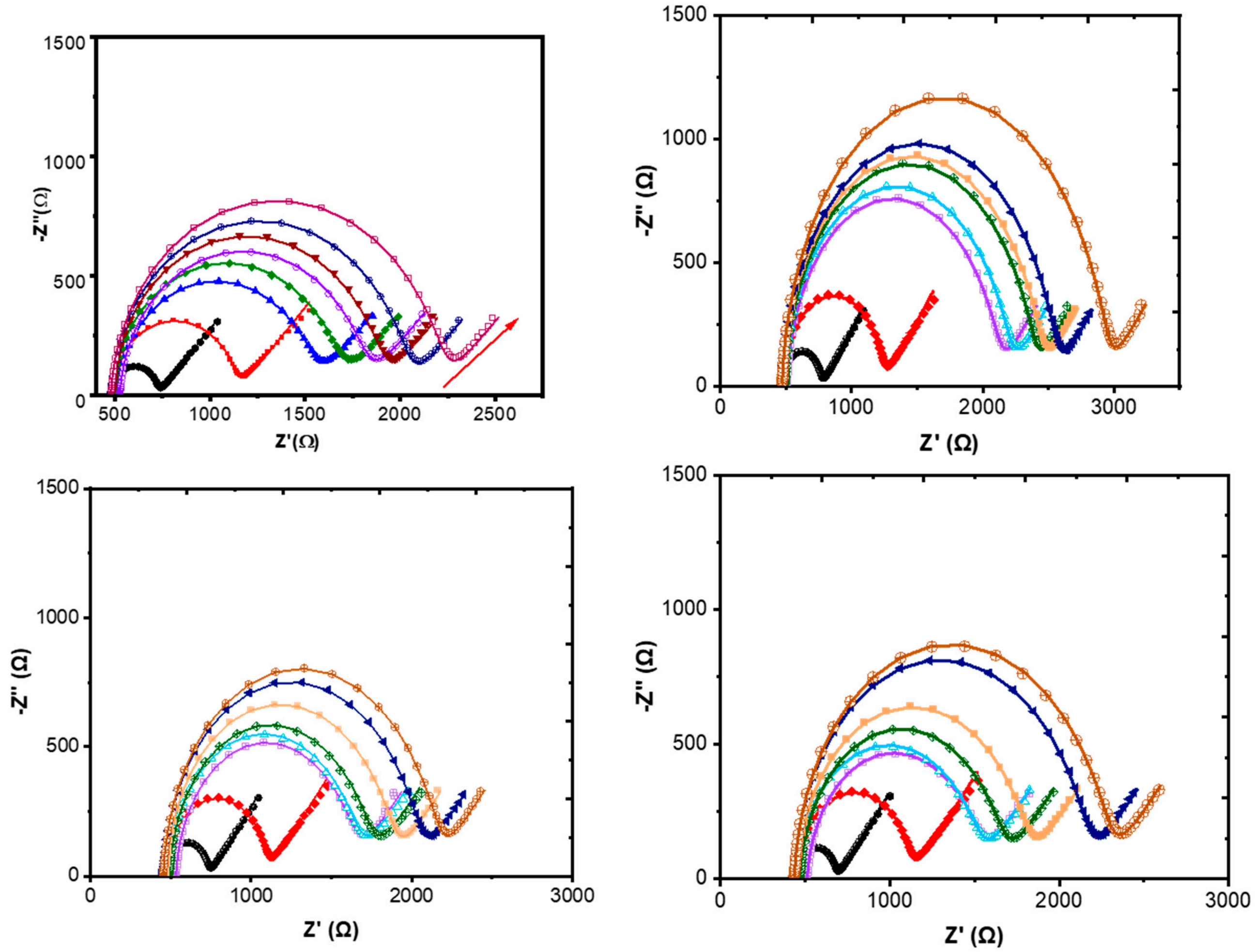

Figure S5. Nyquist diagrams of a SA-PEO/FA-modified SPE obtained from increasing concentrations of FRs in plasma for the remaining three electrodes.

Formulation	SA (wt.%)	PEO (wt.%)	SA (wt.%): PEO (wt.%)	FA (mg)	Voltage(kV)	Distance(cm)	Flow rate (mL/h)	Spinnability	Electrospinning process properties	NFs properties
**F1**	3	1.5	70:30	0	11.5	12	0.5	-	Rapid small droplets (no Taylor cone formed)	-
**F2**	3	1.5	70:30	0	13	13.5	0.5	-	Electric discharge problem	-
**F3**	3	1.5	70:30	0	15	13.5	0.5	-	Electric discharge problem	-
**F4**	3	1.5	70:30	0	15	16	0.5	-	Electric discharge problem	-
**F5**	3	1.5	50:50	0	11.5	12	0.5	-	Rapid small droplets (no Taylor cone formed)	-
**F6**	3	1.5	50:50	0	13	13.5	0.5	-	Rapid small droplets (no Taylor cone formed)	-
**F7**	3	1.5	50:50	0	15	13.5	0.5	-	Electric discharge problem	-
**F8**	3	1.5	50:50	0	15	16	0.5	-	Electric discharge problem	-
**F9**	3	1.5	30:70	0	15	13.5	0.5	+	Continuous process with high productivity	Smooth uniform morphology
**F10**	3	1.5	30:70	40	15	13.5	0.5	-	Electric discharge problem	-

**F11**	3	1.5	30:70	20	15	13.5	0.5	+	Continuous process with high productivity	Smooth uniform morphology

Table S1. Composition of the various solutions that were used for the preparation of nanofibers *and the optimization of* the electrospinning process.
Notes: + Fibers formation, - Fibers formation was not observed.

Table S2. Values obtained from the equivalent circuit elements obtained by fitting the EIS experimental data of the SPE and modified SPE with SA-PEO and SA-PEO/FA NFs and the equivalent circuit used for fitting the data.

Element	Rs (Ω)	Rct (Ω)	CPE (μF)	N	W	X^2
**SPCE**	471	674.76	1.21	0.96	0.0026925	9.7222E-4
**(SA-PEO) NFs**	493	381.96	1.28	0.96	0.003019	6.003E-4
**(SA-PEO)/FA NFs**	471	298.07	1.23	0.95	0.0028691	5.6758E-4

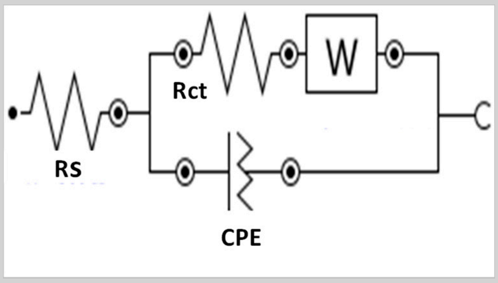

Table S3. Values obtained from the equivalent circuit elements by fitting the EIS experimental data of the biosensor with the incubation of FRs in a buffer of Tris-HCl.

FRs	Rs (Ω)	Rct (Ω)	CPE (μF)	N	W1	X^2
**Blank**	477	252.34	1.08	0.97	0.00288	1.5302E-4
**0.1 PM**	475	474.88	1.17	0.96	0.00238	0.00204
**1 PM**	474	2085.1	2.75	0.94	0.00208	0.0039
**10 PM**	480	2436.4	1.93	0.96	0.00255	0.00113
**100 PM**	481	2878.2	1.62	0.96	0.00252	0.00127
**1 nM**	479	2959.3	1.46	0.96	0.00246	0.00159

Table S4. The selectivity of the developed biosensor for FRs by fitting the EIS experimental data **in** $[Fe(CN)_6]^{3-/4-}$ after incubating the NF-modified electrodes with 10 nM FRs (target) for 1hr, 100 nM HSA for 1 hr, and a mixture of both proteins (10 nM FRs+ 100 nM HSA) for 1hr.

Element	Rs (Ω)	Rct (Ω)	CPE (µF)	N	W1	X^2	ΔR
**SA-PEO/FA NFs (blank)**	458	167	13.3	0.748	0.0028	7.141E-4	……
**SA-PEO/FA NFs/ FRs**	453	546.61	5.54	0.847	0.0025	0.0075927	2.27

Element	Rs (Ω)	Rct (Ω)	CPE (µF)	N	W1	X^2	ΔR
**SA-PEO/FA NFs (blank)**	450	157.92	4.16	0.86	0.0029	3.1021E-4	……
**SA-PEO/FA NFs/ HSA**	451	167.63	1.26	0.95	0.0028	4.6672E-4	0.061

Element	Rs (Ω)	Rct (Ω)	CPE (µF)	N	W1	X^2	ΔR
**SA-PEO/FA NFs (blank)**	472	161.4	11.1	0.76	0.002835	0.0011833	…..
**SA-PEO/FA NFs+ (FRs, HSA)**	463	501.61	7.52	0.81	0.00262	0.0066175	2.1

Table S5. The stability of the NF biosensor by fitting the EIS experimental data **in** $[Fe(CN)_6]^{3-/4-}$ after storing the NF modified electrode for two months at 4 °C.

Element	Rs (Ω)	Rct (Ω)	CPE (µF)	C (nF)	N	W1	X^2
**(SA-PEO)/FA NFs freshly prepared**	497	247.06	1.24	……	0.96	0.0029518	4.4989E-4
**(SA-PEO)/FA NFs after 2 months**	501	228.22	……..	934	…...	0.0028747	0.0011803

Table S6. Values obtained from the equivalent circuit elements by fitting the EIS experimental data of the biosensor measured in human plasma.

FRs	Rs (Ω)	Rct (Ω)	CPE (μF)	N	W1	X^2
**Blank**	477.85	246.35	1.14	0.97	0.0028	2.114E-4
**0.1 PM**	482.77	651.58	1.39	0.96	0.0023	0.0027147
**1 PM**	511.48	1014.4	5.45	0.95	0.0026	3.6599E-4
**10 PM**	487.33	1173.6	4.68	0.95	0.0026	5.9686E-4
**100 PM**	526.49	1265.6	3.98	0.96	0.0026	3.8985E-4
**1 nM**	485.47	1394.8	3.26	0.96	0.0028	0.0011399
**10 nM**	477.88	1540.4	2.41	0.96	0.0028	6.835E-4
**100 nM**	478.93	1720.5	2.35	0.96	0.0028	0.0010709

Determination of the constant of heterogenous electron transfer from Rct.

When we have a redox couple in the solution that could make a redox reaction on the surface, the direct relation between the current and the potential is related to the Butler–Volmer equation, which is related to the amount of the electron transfer and applicable when the polarization depends on the charge transfer kinetic.

$$i_0 = \mathrm{nF}k_0 Cexp - \alpha nF\frac{\left(E_{app} - E_{ocp}\right)}{RT}$$

Butler–Volmer equation

where i_0 is the current exchange density, F is the Faraday constant, n is the number of electrons involved, C is the concentration of the redox species, k_0 is the heterogenous charge transfer, (E_{app}-E_{ocp}) is the overpotential, and α is the coefficient of the electron transfer.

When the over potential is very low, the current exchange is related to the charge transfer resistance and the Butler–Volmer equation is simplified, giving the two following equations.

$$i_o = \frac{RT}{nF}\frac{1}{R_{ct}} \qquad\qquad i_0 = nFACk_0$$

The heterogenous charge transfer (k_0) could be calculated following the two equations.

$$k_0 = \frac{RT}{n^2F^2CAR_{ct}}$$
